# Association of Combined Nutritional Risk and Oral Frailty Assessment with Postoperative Complications in Older Surgical Patients: A Pilot Cohort Study

**DOI:** 10.31662/jmaj.2025-0520

**Published:** 2026-07-03

**Authors:** Hiroyasu Murata

**Affiliations:** 1 Department of Physical Therapy, Faculty of Health Science, Kyorin University, Tokyo, Japan

**Keywords:** nutritional risk, oral frailty, postoperative complications, older adults, surgery

## Abstract

**Introduction:**

Postoperative complications are common in older adults and are influenced by frailty-related factors. This study examined whether the combined assessment of nutritional risk and oral frailty was associated with postoperative complications in older surgical patients.

**Methods:**

We retrospectively analyzed 200 patients aged ≥65 years who underwent elective surgery under general anesthesia at Kyorin University Hospital (July–October 2021). Nutritional risk was defined as a Geriatric Nutritional Risk Index (GNRI) <98, and oral frailty as an Oral Frailty Index-8 score ≥4. Patients were stratified into four groups: no risk, oral frailty only, nutritional risk only, and both conditions. The primary outcome was complications of Clavien–Dindo grade ≥II. Multivariable logistic regression analyses were performed, including a sensitivity analysis using log-transformed intraoperative blood loss as an alternative indicator of surgical invasiveness.

**Results:**

Median age was 75 years (interquartile range 70-80), and 52.5% were male. Complications occurred in 32.0% overall: 22.4% (no risk), 25.9% (oral frailty only), 45.0% (nutritional risk only), and 50.0% (both conditions) (p < 0.01). In multivariable models adjusted for age, sex, and American Society of Anesthesiologists Physical Status, nutritional risk (adjusted odds ratio [aOR] 2.72, 95% confidence interval [CI] 1.13-6.51) and combined risk (aOR 2.85, 95% CI 1.12-7.23) were significant predictors. After additional adjustment for operative time, only nutritional risk remained significant (aOR 2.86, 95% CI 1.11-7.36), whereas in a sensitivity analysis using log-transformed intraoperative blood loss, both nutritional risk alone (aOR 3.12, 95% CI 1.21-8.01) and combined risk (aOR 3.26, 95% CI 1.21-8.75) remained significantly associated with complications.

**Conclusions:**

Preoperative nutritional risk assessed by the GNRI was consistently associated with postoperative complications, whereas oral frailty alone was not. Although the combined presence of nutritional risk and oral frailty showed the highest crude complication burden, its association varied according to the indicator of surgical invasiveness. These findings support the importance of nutritional assessment in perioperative risk stratification in older surgical patients.

## Introduction

Postoperative complications are a leading cause of morbidity, mortality, prolonged hospitalization, and increased healthcare costs in older adults ^(1)^. Conventional risk assessments emphasize comorbidities and organ function, but functional decline, nutrition, and oral health are increasingly recognized as crucial determinants of surgical outcomes ^(2), (3), (4)^. Among them, nutritional risk and oral frailty represent potentially modifiable factors that can be readily evaluated in routine care.

Nutritional risk strongly influences surgical prognosis. The Geriatric Nutritional Risk Index (GNRI) has been widely validated, with low scores associated with infections, delayed healing, and longer hospital stays ^(5), (6)^. Oral frailty, defined as age-related decline in chewing and swallowing, also contributes to poor outcomes ^(4)^. Screening tools such as the Oral Frailty Index-8 (OFI-8) are practical for bedside use ^(7)^, and high scores correlate with reduced food intake and greater risk of disability ^(8)^. The prevalence of oral frailty among older Japanese individuals is reported to be 38%-55%, which indicates a very high rate ^(9), (10), (11)^.

Despite robust evidence for each factor individually, their combined predictive value for postoperative complications remains unexplored. Most studies have assessed nutrition or oral function in isolation, leaving unclear whether dual assessment improves risk stratification. Moreover, while Japan has pioneered recognition of oral frailty, its integration into perioperative management is limited. Because both indices are simple and low cost, clarifying their combined impact could establish a more comprehensive and practical approach to preoperative evaluation.

This pilot observational cohort study aimed to examine the association of nutritional risk and oral frailty, individually and in combination, with postoperative complications in older Japanese surgical patients. We hypothesized that patients with both nutritional risk and oral frailty would have a higher risk of postoperative complications than those with either condition alone or neither condition. By analyzing older Japanese surgical patients, we sought to generate population-specific evidence to support a simple and practical approach to perioperative risk assessment.

## Materials and Methods

### Study design and patient recruitment

This retrospective observational cohort study included patients aged ≥65 years who underwent elective surgery under general anesthesia at Kyorin University Hospital (Tokyo, Japan). All patients were referred to the Perioperative Management Center and underwent preoperative evaluation by anesthesiologists. The study period was between July 5 and October 1, 2021, based on the date of preoperative anesthesiology consultation. A total of 200 patients were included. Patients who were unable to complete the preoperative interview because of cognitive or communication difficulties were excluded.

### Ethics approval and informed consent

The study protocol was approved by the Ethics Committee of Kyorin University School of Medicine (approval number: R04-012). Given the retrospective design, the requirement for written informed consent was waived, with an opt-out approach applied. All procedures were conducted in accordance with the principles of the Declaration of Helsinki.

### Baseline patient characteristics

Patient background information included age, sex, body mass index (BMI), American Society of Anesthesiologists Physical Status (ASA-PS) ^(12)^, smoking history, presence of comorbidities, medications, blood collection data, and surgical data.

### Assessment of nutritional risk

Nutritional risk was evaluated using the GNRI. It was calculated as follows ^(13)^: GNRI = 1.489 × albumin (g/L) + 41.7 × present body weight/ideal body weight, which is equivalent to 1.489 × albumin (g/L) + 41.7 × BMI/22. In accordance with previous literature, GNRI <98 was classified as nutritional risk, whereas GNRI ≥98 indicated no nutritional risk ^(14)^.

### Assessment of oral frailty

Oral frailty was assessed using the OFI-8, a validated screening tool based on eight self-reported items, including chewing, swallowing, and oral health-related functions. A score ≥4 was defined as oral frailty, while scores ≤3 indicated nonfrailty ^(7)^.

### Group classification

Patients were stratified into four groups according to the presence or absence of nutritional risk and oral frailty:

•Group 1: No nutritional risk and no oral frailty

•Group 2: Oral frailty only

•Group 3: Nutritional risk only

•Group 4: Both nutritional risk and oral frailty

### Outcomes

The primary outcome was the incidence of postoperative complications during hospitalization, defined as Clavien–Dindo grade ≥II ^(15)^. Complications were identified and evaluated by attending surgeons or anesthesiologists.

### Statistical analysis

Categorical variables were compared among groups using the chi-square test, with post hoc residual analysis to explore intergroup differences. Logistic regression analysis was performed to examine the association between group classification and postoperative complications. Model 1 was unadjusted. Model 2 was adjusted for age, sex, and ASA-PS. Model 3 additionally included operative time as an indicator of surgical invasiveness. Because intraoperative blood loss showed a skewed distribution, it was log-transformed as ln(blood loss + 1), and a sensitivity analysis was performed using this variable instead of operative time (Model 4). Results were expressed as odds ratios (ORs) with 95% confidence intervals (CIs). Statistical significance was defined as p < 0.05. All analyses were conducted using EZR (Saitama Medical Center, Jichi Medical University, Saitama, Japan), a graphical user interface for R (R Foundation for Statistical Computing, Vienna, Austria) [3].

## Results

### Patient characteristics

A total of 200 patients aged ≥65 years who underwent elective surgery under general anesthesia were included. The median age was 75 years (interquartile range 70-80), and 105 (52.5%) were male. Baseline demographics and clinical characteristics are summarized in Table 1. Patients were classified into four groups: no nutritional risk and no oral frailty (Group 1, n = 76), oral frailty only (Group 2, n = 54), nutritional risk only (Group 3, n = 40), and both nutritional risk and oral frailty (Group 4, n = 30). Between-group comparisons and standardized mean differences are shown in Table 2. Significant differences were observed in age, sex distribution, BMI, ASA-PS, surgical category, and operative time (all p < 0.05), whereas intraoperative blood loss did not differ significantly among the groups.

**Table 1.** Baseline Characteristics of All Patients (n = 200).

Characteristics		Values
Age (years), median (IQR)		75 (70-80)
Sex: male, n (%)		105 (52.5)
BMI (kg/m^2^), median (IQR)		22.5 (19.6-24.9)
ASA-PS (1/2/3/4), n (%)		4 (2.0)/151 (75.5)/43 (21.5)/2 (1.0)
Smoking (never/past/current), n (%)		90 (45.0)/88 (44.0)/22 (11.0)
Surgical category, n (%)		
	Gastroenterological surgery	43 (21.5)
	Thoracic surgery	22 (11.0)
	Urology	34 (17.0)
	Orthopedics	37 (18.5)
	Gynecology	14 (7.0)
	Other (plastic reconstructive, otolaryngology–head and neck surgery, cardiovascular, ophthalmology, breast, neurosurgery, and dermatology)	50 (25.0)
Presence of comorbidities, n (%)		
	Hypertension	116 (58.0)
	Diabetes	48 (24.0)
	Coronary artery disease	19 (9.5)
	Dyslipidemia	65 (32.5)
	Liver dysfunction	14 (7.0)
	Renal insufficiency	155 (77.5)
	Heart failure	3 (1.5)
	Atrial fibrillation	10 (5.0)
	ASO	4 (2.0)
	Cerebral infarction	16 (8.0)
	COPD	9 (4.5)
Medications, n (%)		
	Insulin	29 (14.5)
	Oral hypoglycemic agents	97 (48.5)
	Antihypertensive agents	11 (5.5)
	Diuretics	42 (21.0)
	Antiplatelet agents	23 (11.5)
	Anticoagulants	11 (5.5)
	Bronchodilators	8 (4.0)
	Steroids	43 (21.5)
	Antipsychotics	22 (11.0)
Blood collection data, median (IQR)		
	WBC count (×10^3^/μL)	5.80 (4.50-7.03)
	RBC count (×10^6^/μL)	4.14 (3.76-4.52)
	Hemoglobin (g/dL)	13.1 (11.9-14.1)
	Hematocrit (%)	39.1 (35.6-42.0)
	Platelets (×10^4^/μL)	22.7 (18.9-27.4)
	AST (IU/L)	21.5 (18.0-27.0)
	ALT (IU/L)	17.0 (12.0-24.0)
	BUN (mg/dL)	16.3 (13.5-20.1)
	Creatinine (mg/dL)	0.79 (0.65-0.98)
	eGFR (mL/min/1.73 m^2^)	63.5 (52.3-73.8)
	CRP (mg/dL)	0.08 (0.04-0.31)
	Total protein (g/dL)	7.05 (6.70-7.40)
	Albumin (g/dL)	4.10 (3.80-4.30)
Surgical data		
	Operative time (min), median (IQR)	166.5 (108.8-246.8)
	Anesthesia time (min), median (IQR)	237.0 (163.8-322.3)
	Epidural anesthesia, n (%)	53 (26.5)
	Spinal anesthesia, n (%)	1 (0.5)
	Peripheral nerve block, n (%)	51 (25.5)
	Intraoperative fluid administration (mL), median (IQR)	1125.0 (750.0-1812.5)
	Intraoperative blood loss (mL), median (IQR)	34.0 (5.0-170.3)
	Intraoperative blood transfusion, n (%)	11 (5.5)
GNRI, median (IQR)		102.7 (95.5-109.5)
GNRI <98, n (%)		70 (35.0)
OFI-8 score, median (IQR)		3 (2-5)
OFI-8 ≥4, n (%)		84 (42.0)
Postoperative LOS, days, median (IQR)		9 (6-16)
Complications, Clavien–Dindo grade ≥II, n (%)		64 (32.0)
Clavien–Dindo grade, n (%)		
	I	21 (10.5)
	II	49 (24.5)
	IIIa	6 (3.0)
	IIIb	3 (1.5)
	IVa	3 (1.5)
	IVb	1 (0.5)
	V	2 (1.0)

ALT: alanine aminotransferase; ASA-PS: American Society of Anesthesiologists Physical Status; ASO: arteriosclerosis obliterans; AST: aspartate aminotransferase; BMI: body mass index; BUN: blood urea nitrogen; COPD: chronic obstructive pulmonary disease; CRP: C-reactive protein; eGFR: estimated glomerular filtration rate; GNRI: Geriatric Nutritional Risk Index; IQR: interquartile range; LOS: length of stay; OFI-8: Oral Frailty Index-8; RBC: red blood cell; WBC: white blood cell.

**Table 2.** Baseline Characteristics of Patients Stratified by Preoperative Nutritional and Oral Functional Status.

Variables		Group 1 (n=76)	Group 2 (n=54)	Group 3 (n=40)	Group 4 (n=30)	SMD	p-Value
Age (years), median (IQR)		73 (69-77)	79 (74-81)	75 (70-81)	76 (71-80)	0.77	<0.01
Sex: male, n (%)		46 (60.5)	29 (53.7)	10 (25.0)	20 (66.7)	0.84	<0.01
BMI, kg/m^2^, median (IQR)		24.0 (22.1-25.9)	23.5 (21.7-25.8)	18.1 (16.7-20.1)	19.1 (17.7-23.1)	1.82	<0.01
ASA-PS (1/2/3/4), n (%)		1 (1.3)/68 (89.5)/6 (7.9)/1 (1.3)	2 (3.7)/38 (70.4)/13 (24.1)/1 (1.9)	0 (0)/30 (75.0)/10 (25.0)/0 (0)	1 (3.3)/15 (50.0)/14 (46.7)/0 (0)	0.99	<0.01
Smoking (never/past/current), n (%)		32 (42.1)/38 (50.0)/6 (7.9)	24 (44.4)/24 (44.4)/6 (11.1)	25 (62.5)/12 (30.0)/3 (7.5)	9 (30.0)/14 (46.7)/7 (23.3)	0.47	0.06
Surgical category, n (%)						0.59	0.03
	Gastroenterological surgery	14 (18.4)	11 (20.4)	8 (20.0)	10 (33.3)		
	Thoracic surgery	13 (17.1)	4 (7.4)	3 (7.5)	2 (6.7)		
	Urology	13 (17.1)	12 (22.2)	6 (15.0)	3 (10.0)		
	Orthopedics	14 (18.4)	14 (25.9)	6 (15.0)	3 (10.0)		
	Gynecology	2 (2.6)	5 (9.3)	7 (17.5)	0 (0.0)		
	Other (plastic reconstructive, otolaryngology-head and neck surgery, cardiovascular, ophthalmology, breast, neurosurgery, and dermatology)	20 (26.3)	8 (14.8)	10 (25.0)	12 (40.0)
Operative time (min), median (IQR)		161.5 (97.8-234.8)	157.5 (111.5-258.8)	144.5 (104.5-246.8)	217.0 (190.8-354.8)	0.69	0.03
Intraoperative blood loss (mL), median (IQR)		26.0 (3.0-158.3)	58.0 (8.0-165.0)	27.5 (4.0-172.8)	37.0 (10.3-165.0)	0.22	0.68

ASA-PS: American Society of Anesthesiologists Physical Status; BMI: body mass index; IQR: interquartile range; SMD: standardized mean difference.

### Incidence of postoperative complications

Overall, 64 patients (32.0%) developed postoperative complications of Clavien–Dindo grade ≥II during hospitalization (Table 3). The incidence of complications was as follows:

**Table 3.** Incidence of Postoperative Complications in the Four Groups Classified by GNRI and OFI-8.

Groups	Complications, Clavien–Dindo grade ≥II, n (%)	Standardized residuals
Group 1 (n = 76)	17 (22.4)	−1.48
Group 2 (n = 54)	14 (25.9)	−0.79
Group 3 (n = 40)	18 (45.0)	+1.45
Group 4 (n = 30)	15 (50.0)	+1.74

Notes: Chi-square test: χ^2^ = 11.73, df = 3; p < 0.01; standardized residuals with |z| > 1.96 were considered statistically significant. GNRI: Geriatric Nutritional Risk Index; OFI-8: Oral Frailty Index-8.

•Group 1: 22.4%

•Group 2: 25.9%

•Group 3: 45.0%

•Group 4: 50.0%

The chi-square test revealed a significant difference in complication rates among the four groups (χ^2^ = 11.73, df = 3; p < 0.01). Standardized residual analysis showed a numerical tendency toward higher complication rates in Groups 3 and 4 and toward a lower complication rate in Group 1, although none of the residuals exceeded the threshold for statistical significance.

### Association between group classification and complications

Univariate logistic regression showed that both nutritional risk only (OR 2.84, 95% CI 1.25-6.47, p = 0.01) and combined nutritional risk with oral frailty (OR 3.47, 95% CI 1.42-8.50, p = 0.01) were significantly associated with postoperative complications, while oral frailty alone was not (OR 1.21, 95% CI 0.54-2.74, p = 0.64).

In multivariable logistic regression adjusted for age, sex, and ASA-PS, both nutritional risk only (adjusted OR 2.72, 95% CI 1.13-6.51, p = 0.03) and the combination of nutritional risk and oral frailty (adjusted OR 2.85, 95% CI 1.12-7.23, p = 0.03) remained significant predictors of postoperative complications, while oral frailty alone did not (Table 4, Model 2). ASA-PS was also an independent predictor (adjusted OR 1.95, 95% CI 1.02-3.74, p = 0.04).

**Table 4.** Multivariate Logistic Regression Analysis of Factors Associated with Postoperative Complications.

Variables	Model 1		Model 2		Model 3		Model 4	
	Crude OR (95% CI)	p-Value	aOR (95% CI)	p-Value	aOR (95% CI)	p-Value	aOR (95% CI)	p-Value
Group 1	Ref	Ref	Ref	Ref	Ref	Ref	Ref	Ref
Group 2	1.21 (0.54 -2.74)	0.64	1.14 (0.48-2.71)	0.76	1.12 (0.45-2.79)	0.82	1.02 (0.42-2.51)	0.96
Group 3	2.84 (1.25 -6.47)	<0.05	2.72 (1.13-6.51)	0.03	2.86 (1.11-7.36)	0.03	3.12 (1.21-8.01)	0.02
Group 4	3.47 (1.42 -8.50)	<0.01	2.85 (1.12-7.23)	0.03	1.99 (0.71-5.55)	0.19	3.26 (1.21-8.75)	0.02
Age	NA	NA	0.99 (0.94-1.04)	0.70	1.00 (0.94-1.06)	0.90	1.00 (0.94-1.05)	0.91
Sex	NA	NA	0.93 (0.48-1.78)	0.82	1.07 (0.53-2.18)	0.82	0.85 (0.43-1.71)	0.66
ASA-PS	NA	NA	1.95 (1.02-3.74)	0.04	1.68 (0.84-3.38)	0.14	1.38 (0.68-2.80)	0.38
Operative time	NA	NA	NA	NA	1.01 (1.00-1.01)	<0.01	−	−
Log-transformed intraoperative blood loss	NA	NA	NA	NA	NA	NA	1.44 (1.21-1.72)	<0.01

Notes: Model 1 was unadjusted; Model 2 was adjusted for age, sex, and ASA-PS; Model 3 was additionally adjusted for operative time; Model 4 was adjusted for age, sex, ASA-PS, and log-transformed intraoperative blood loss (ln[blood loss + 1]) as a sensitivity analysis; OR for operative time is expressed per 1-minute increase; OR for log-transformed intraoperative blood loss is expressed per 1-unit increase (ln[blood loss + 1]). aOR: adjusted odds ratio; ASA-PS: American Society of Anesthesiologists Physical Status; CI: confidence interval; OR: odds ratio.

When operative time was additionally included in the model, nutritional risk only remained significant (adjusted OR 2.86, 95% CI 1.11-7.36, p = 0.03), whereas the combination of nutritional risk and oral frailty was no longer significant (adjusted OR 1.99, 95% CI 0.71-5.55, p = 0.19). Operative time itself was strongly associated with complications (adjusted OR 1.01 per minute, 95% CI 1.00-1.01, p < 0.01) (Table 4, Model 3).

In a sensitivity analysis replacing operative time with log-transformed intraoperative blood loss, both nutritional risk only (adjusted OR 3.12, 95% CI 1.21-8.01, p = 0.02) and the combination of nutritional risk and oral frailty (adjusted OR 3.26, 95% CI 1.21-8.75, p = 0.02) remained significantly associated with postoperative complications. Log-transformed intraoperative blood loss was also independently associated with postoperative complications (adjusted OR 1.44, 95% CI 1.21-1.72, p < 0.01) (Table 4, Model 4).

## Discussion

In this pilot observational cohort study of 200 older adults undergoing elective surgery under general anesthesia, preoperative nutritional risk assessed by the GNRI was consistently associated with postoperative complications. Patients with nutritional risk alone had an approximately threefold higher risk of complications compared with those with neither nutritional risk nor oral frailty, even after adjustment for age, sex, ASA-PS, and alternative indicators of surgical invasiveness. In contrast, oral frailty assessed by the OFI-8 alone was not independently associated with complications. Although the coexistence of nutritional risk and oral frailty was associated with the highest crude incidence of complications (50.0%), its association was attenuated after adjustment for operative time but remained significant in a sensitivity analysis using log-transformed intraoperative blood loss. These findings suggest that different intraoperative indicators may capture distinct aspects of surgical stress or procedural complexity, rather than indicating that oral frailty is clinically irrelevant. To our knowledge, this is the first study to directly evaluate the combined role of the GNRI and OFI-8 in relation to postoperative complications in a Japanese perioperative cohort.

Our findings are in line with previous reports that preoperative nutritional status is a strong predictor of adverse surgical outcomes. Several studies have demonstrated that a low GNRI is associated with increased rates of postoperative complications, longer hospital stays, and higher mortality across various surgical populations, including gastrointestinal, cardiovascular, and orthopedic surgeries ^(5), (6), (16), (17)^. In particular, GNRI <98 has been widely recognized as a clinically relevant cutoff for defining nutritional risk in older adults ^(18), (19)^. Our study corroborates these observations, showing that nutritional risk alone nearly tripled the risk of complications even after adjustment for confounding factors. In contrast, evidence regarding oral frailty as an independent perioperative risk factor remains limited. Previous Japanese cohort studies have reported that reduced oral function, such as impaired mastication and swallowing, is associated with poor nutritional intake and sarcopenia, which in turn may predispose patients to postoperative morbidity ^(20), (21), (22)^. However, oral frailty itself was not significantly associated with complications in our analysis. This discrepancy may be explained by differences in study populations, oral frailty assessment tools, and perioperative oral care protocols. Interestingly, although the coexistence of nutritional risk and oral frailty was associated with the highest crude complication rate, this association was attenuated after adjustment for operative time, whereas it remained significant in the sensitivity model using log-transformed intraoperative blood loss. This pattern suggests that operative time and blood loss may capture different dimensions of surgical invasiveness. Previous reports have also emphasized that longer operative time and increased surgical complexity are among the strongest intraoperative predictors of complications ^(23), (24), (25)^. Therefore, the attenuation observed in the operative time-adjusted model may indicate that operative time reflects broader procedural complexity and cumulative physiological stress, rather than simply implying that oral frailty is a proxy for complex surgery or that surgical stress completely outweighs oral function. In contrast, oral frailty may represent a contributory vulnerability whose effect becomes more clinically relevant when combined with poor nutritional status. However, in our cohort, oral frailty alone was not significantly associated with postoperative complications. One possible explanation is the implementation of perioperative oral management at our institution. Indeed, a previous study from our hospital demonstrated that an innovative oral management procedure, emphasizing oral mucosa stimulation to promote saliva discharge, significantly reduced the risk of postoperative pneumonia and complications in patients undergoing lung cancer surgery ^(26)^. This proactive approach may partly explain why oral frailty alone was not significantly associated with postoperative complications in our cohort. These findings suggest that while oral frailty represents a risk factor in general, institutional practices in perioperative oral management may influence its clinical impact.

Several mechanisms may explain the observed association between nutritional risk and postoperative complications. First, malnutrition impairs immune function, delays wound healing, and increases susceptibility to infections, all of which contribute to higher rates of postoperative morbidity ^(27), (28)^. The GNRI, which incorporates serum albumin levels and body weight relative to ideal body weight, reflects both nutritional and inflammatory status. A lower GNRI therefore captures a state of diminished physiological reserve, predisposing patients to adverse outcomes after surgery. Second, nutritional deficits are closely linked to sarcopenia, which reduces skeletal muscle mass and strength. Sarcopenia has been reported to impair respiratory function, increase the risk of pneumonia, and prolong recovery after surgery ^(29), (30)^. In our cohort, patients with nutritional risk likely had decreased functional reserve, making them more vulnerable to surgical stress and complications. Regarding oral frailty, previous studies have shown that impaired oral function—such as reduced mastication, tongue pressure, and swallowing—can lead to inadequate dietary intake, aspiration, and poorer nutritional status ^(20), (31)^. While oral frailty alone was not an independent risk factor in our analysis, it may contribute indirectly to adverse outcomes through its synergistic interaction with malnutrition. The finding that the combination of oral frailty and nutritional risk was associated with the highest crude complication rate supports this possibility. Finally, surgical invasiveness, reflected by longer operative time, may exacerbate these vulnerabilities. Prolonged surgery is associated with increased blood loss, fluid shifts, hypothermia, and systemic inflammation ^(32), (33)^, all of which amplify the impact of preoperative frailty states on postoperative outcomes. Thus, our findings suggest that nutritional risk represents a direct and robust predictor of postoperative complications, whereas oral frailty acts as a contributory factor whose impact is magnified in the context of higher surgical stress.

Our findings underscore the importance of preoperative nutritional screening in older adults undergoing elective surgery. GNRI <98 identified a subgroup of patients with a substantially increased risk of postoperative complications, even in the absence of oral frailty. This supports the integration of the GNRI into routine perioperative assessment as a simple and objective tool that can be calculated from serum albumin levels and body weight, which are already obtained in standard preoperative evaluations. Although oral frailty alone was not independently predictive, its coexistence with nutritional risk was associated with the highest crude complication rate and remained significant in the blood loss–adjusted sensitivity analysis. This suggests that a combined assessment of nutritional and oral status may help identify patients with increased perioperative vulnerability. In clinical practice, such patients might benefit from multidisciplinary interventions combining nutritional support and preoperative oral management. Furthermore, the attenuation of the effect of combined risk after adjustment for operative time highlights the interplay between patient frailty and surgical invasiveness. These results emphasize the need for individualized perioperative management strategies that consider not only patient-related factors but also modifiable surgical factors. Prehabilitation programs addressing nutrition and oral health, alongside careful surgical planning to minimize operative stress, may collectively reduce postoperative morbidity in older adults ^(34)^.

Several limitations of this study should be acknowledged. First, the retrospective and single-center design may introduce selection bias and limit the generalizability of the findings. Our sample was drawn from a perioperative cohort in a university hospital setting, and the results may not be directly applicable to other surgical populations or healthcare systems. Second, although we adjusted for major confounders, including age, sex, ASA-PS, and indicators of surgical invasiveness, residual confounding cannot be excluded. In particular, surgical category differed across groups, and disease-specific factors such as tumor stage, surgical technique, and anesthetic management were not fully accounted for. We additionally performed a sensitivity analysis using log-transformed intraoperative blood loss; however, this does not eliminate the possibility of residual confounding related to procedural complexity. Intraoperative transfusion was available in the dataset, but the small number of transfused cases limited its use in multivariable adjustment. Third, the assessment of oral frailty was based on the OFI-8 questionnaire, which may not fully capture the complexity of oral function, such as masticatory performance or tongue pressure. More comprehensive oral assessments might have provided greater sensitivity in detecting the impact of oral health on outcomes. Finally, the sample size was relatively small, particularly for subgroup analyses, which may have reduced statistical power to detect moderate associations. This limitation highlights the need for larger, multicenter, prospective studies to validate these findings.

### Conclusions

In this pilot observational cohort study of older adults undergoing elective surgery, preoperative nutritional risk assessed by the GNRI was identified as a robust predictor of postoperative complications. Patients with nutritional risk alone had a significantly elevated risk, whereas oral frailty alone was not independently associated with adverse outcomes. Although the coexistence of nutritional risk and oral frailty showed attenuation after adjustment for operative time, it remained significantly associated with complications in a sensitivity analysis using log-transformed intraoperative blood loss. These findings suggest that nutritional risk is a consistent predictor of postoperative morbidity and that combined nutritional and oral assessment may help identify patients with heightened perioperative vulnerability. Future multicenter, prospective studies with larger sample sizes are warranted to validate these results and to determine whether targeted perioperative interventions can reduce postoperative complications in older surgical populations.

## Article Information

### Acknowledgments

I thank the medical and nursing staff of the Perioperative Management Center, Kyorin University Hospital, for their dedicated support in patient care and data collection.

### Conflicts of Interest

None

### Ethics Approval

The study protocol was approved by the Ethics Committee of Kyorin University School of Medicine (approval number: R04-012).
